# Supplementary material for: Managing a Curriculum Innovation Process
Source: Pharmacy (Basel). 2020 Aug 24;8(3):153. doi: 10.3390/pharmacy8030153 (PMC7557770; doi:10.3390/pharmacy8030153)
Supplement: Supplementary file 1 [file pharmacy-08-00153-s001.pdf]

Supplemental data

Summary of tools useful to analyse the environment and make sound decisions

PESTEL

This tool focuses mainly on the global macro-environment of an organization and can help to obtain more detailed information on opportunities and threats from the external environment. It stands for:

- **P**olitical – Role of the state and political factors. This is particularly relevant in academia in terms of government funding and the regulations regarding academic liberty and competition.
- **E**conomical – For example, how much students are willing to pay in tuition to get their education during a recession or in a booming economy?
- **S**ocial – What are the demographics and the wealth distribution of the target population (supply and demand)? What is the social status of universities (and of a university degree) in the concerned society?
- **T**echnological – Their impact on academia, course delivery and student expectations. Massive Open Online Courses (MOOC) are good example of the impact of technological influences.
- **E**cological – Sustainable operations and development are expected from all, including Universities and Colleges. This can have an impact on the daily operations.
- **L**egal – What is the regulatory framework of the university or the discipline.

VRIO

Resources and capabilities are the assets of an organization and are necessary to sustain a competitive advantage, to obtain a superior performance or simply to fulfill the mission. They include resources and capabilities in 3 domains: human, physical and financial. The VRIO model helps to analyze these domains and determine the validity of potential solutions when a choice has to be made [1]. It stands for:

- **V**alue of the resources and capabilities – How much do they contribute to help respond to environmental opportunities or threats? How valuable are they to sustain the operations and to satisfy stakeholders?
- **R**arity – How rare are the resources and capabilities and how difficult is it for the competition to possess them?
- **I**nimitability – Are the resources, but most importantly capabilities (specific knowhow, expertise or infrastructure) costly or difficult for others to emulate? Could be in the domains of education, research, outreach or management capabilities.
- **O**rganizational support and knowledge – Is the organization structured in a way that supports the resources and capabilities to provide a distinctive advantage? Does it share its collective intelligence internally to sustain its capabilities?

SWOT

A SWOT analysis provides a general summary of the resources and capabilities (internal) and of the macro-environment (external) for a given organization [1]. The aim is to identify the extent to which strengths and weaknesses (internal factors) influence the response to changes, i.e. opportunities and threats, happening in the external environment. Ideally, a SWOT analysis would be supported by more detailed external (PESTEL) and internal (VRIO) analyses and would allow

to generate possible ideas or solutions to address different combinations of the internal and external factors. SWOT stands for:

- **S**trengths: wide and unique expertise within the College, dedicated staff
- **W**eaknesses: staff inexperienced in curriculum development, high workload, insufficient staff, change-resistant culture
- **O**pportunities: funding, political support for curriculum reform, evolving healthcare system needs
- **T**hreats: opening of a competing university, decreased government funding, restrictive practice regulations

STAKEHOLDER MAPPING

Stakeholders are groups or individuals that depend on the organization to fulfil their own goals or on who the organization depends. A stakeholder map presents the level of interest that they pay towards your organization on the x-axis and the power of the stakeholder to influence the outcome on the y-axis (power-interest matrix) [1]. For each goal or objective, the stakeholder map will vary, as some issues will attract the interest of different stakeholders and they will have variable power to influence the outcome depending on their relationship to the issue.

- **Low interest – Low influence**: devote minimal efforts towards them and simply monitor
- **Low interest – High influence**: keep them satisfied
- **High interest – Low influence**: keep them informed
- **High interest – High influence**: consider them key players, manage closely and include them on key committees

CULTURAL WEB

The cultural web is a tool to analyze the culture of the organization to determine how to best implement the strategy, or to modify some aspects of the strategy (choice of the solutions) to respect the internal culture [1]. It identifies the behavioral, physical and symbolic manifestations of the culture that make up the "taken-for-granted assumptions" of the members of the organization. The cultural web is composed of 7 elements:

- The **paradigm** is "taken-for-granted" assumptions. It is an unconscious bias, mindset or viewpoint that guide decisions and that is based on the below 6 elements.
- **Rituals and routines** are the way of doing things on a day-to-day basis, and the activities that reinforce the culture (training, promotion criteria).
- The **stories** are what is told to new employees when they join to give a sense of what is conventionally important in the organization.
- **Symbols** are related to the rituals and routines, but they are visible manifestations of the culture, like the office layout, the hierarchy-linked benefits and so on.
- The **power** represents the distribution of power within the organization, irrespective of the actual hierarchy. Powerful members are most likely to be enforcing the paradigm.
- **Organizational structures** reflect the importance of control by the management, the participative nature or the collaboration expected within an organization.
- **Control systems are in place to monitor what has importance and relevance to the culture. They include measurements and reward systems like salaries, bonuses and promotions.**

SAFE

There are several tools available to help the decision-making process. One of those is the SAFe tool: it allows to evaluate different options to reduce the gap between the current state and the desired one:

- **S**uitability: Does a proposed solution exploit the key opportunities, while avoiding the major threats? Does it capitalise on the strengths and avoid or improve weaknesses?
- **A**cceptability: Does a proposed solution meet the expectations of stakeholders? Is the expected return acceptable? Is the level of risk acceptable? What will be the reaction of the stakeholders?
- **F**easibility: Does the organization have the capacity to deliver the solution? Are financial resources available or obtainable? Are human resources capable of delivering the solution and, if not, is competent manpower available? Are systems in place (or available) to support it?

Reference:

1. Johnson, G., *et al. Exploring strategy: Text and cases*, 11th ed. Pearson Education Ltd: Harlow, 2017.
